# Use of Echocardiography Reveals Reestablishment of Ventricular Pumping Efficiency and Partial Ventricular Wall Motion Recovery upon Ventricular Cryoinjury in the Zebrafish

**DOI:** 10.1371/journal.pone.0115604

**Published:** 2014-12-22

**Authors:** Juan Manuel González-Rosa, Gabriela Guzmán-Martínez, Inês João Marques, Héctor Sánchez-Iranzo, Luis Jesús Jiménez-Borreguero, Nadia Mercader

**Affiliations:** 1 Department of Cardiovascular Development and Repair, Atherothrombosis and Imaging, Centro Nacional de Investigaciones Cardiovasculares (CNIC), Madrid, Spain; 2 Department of Epidemiology, Atherothrombosis and Imaging, Centro Nacional de Investigaciones Cardiovasculares (CNIC), Madrid, Spain; 3 Department of Cardiology, University Hospital La Paz, Madrid, Spain; 4 Hospital de La Princesa, Madrid, Spain; University of Sheffield, United Kingdom

## Abstract

**Aims:**

While zebrafish embryos are amenable to in vivo imaging, allowing the study of morphogenetic processes during development, intravital imaging of adults is hampered by their small size and loss of transparency. The use of adult zebrafish as a vertebrate model of cardiac disease and regeneration is increasing at high speed. It is therefore of great importance to establish appropriate and robust methods to measure cardiac function parameters.

**Methods and Results:**

Here we describe the use of 2D-echocardiography to study the fractional volume shortening and segmental wall motion of the ventricle. Our data show that 2D-echocardiography can be used to evaluate cardiac injury and also to study recovery of cardiac function. Interestingly, our results show that while global systolic function recovered following cardiac cryoinjury, ventricular wall motion was only partially restored.

**Conclusion:**

Cryoinjury leads to long-lasting impairment of cardiac contraction, partially mimicking the consequences of myocardial infarction in humans. Functional assessment of heart regeneration by echocardiography allows a deeper understanding of the mechanisms of cardiac regeneration and has the advantage of being easily transferable to other cardiovascular zebrafish disease models.

## Introduction

Adult mammalian cardiomyocytes possess a limited capacity for self-renewal in homeostatic and pathologic situations [1], [2]. Myocardial infarction (MI), the most common cause of cardiac injury in humans, results in acute loss of large numbers of cardiomyocytes that are substituted by scar tissue [3]. Although this scar provides mechanical support to the infarcted heart, preventing wall rupture during the post-infarction period, scarring progressively leads to changes in ventricular geometry, referred to as “ventricular remodeling”, which can ultimately lead to cardiac failure. In contrast, other vertebrates, such as teleost fish, have a marked capacity to regenerate cardiac tissue after injury in the adult. The zebrafish (*Danio rerio*) is a well-established model system to study cardiac regeneration. Examination of the mechanisms underlying heart regeneration in the zebrafish may lead to the identification of novel strategies to improve the limited regenerative response in mammals [4]. Models of cardiac insult in the zebrafish include ventricular resection [5], [6] in addition to strategies that induce cell death rather than tissue removal, such as ventricular cryoinjury [7]–[9] or genetic ablation of cardiomyocytes [10].

Although the structural recovery of ventricular organization occurs following any of these injury methods, the recovery of the electrical and mechanical heart functions has been only partially examined. Accordingly, optical mapping has been used to study the electrical coupling of newly formed cardiomyocytes after ventricular resection, with results suggesting that cardiac conduction is completely recovered [11]. Electrocardiography has also been used to assess electrical coupling after ventricular resection or cryoinjury [7], [12], [13]. This method revealed a correlation between QTc prolongation and J-point depression with cardiac injury. At later stages the correlation was not statistically significant, suggesting a recovery of cardiac conduction. An exercise test, in which animals are placed in a swim tube with a tunable water flow velocity, has allowed the study of swimming performance as an indirect metric of cardiac mechanical function upon genetic ablation of cardiomyocytes [10]. Finally, cardiac function has been also evaluated using genetic engineering protocols, whereby a transgenic line, a nearly transparent *casper* mutant, carries the *myl7*:DsRed transgene and expresses red fluorescent protein in the myocardium [14]. While optical mapping and electrocardiography are valuable tools to study electrical coupling of cardiomyocytes *ex vivo* and *in vivo*, respectively, unfortunately, they do not provide information on cardiac pumping efficiency. Moreover, the relationship between QT interval and myocardial infarction and its use as a predictor in electrophysiological studies in patients with coronary artery disease, is controversial [15], [16]. Similarly, the exercise swim test provides only indirect evidence of cardiac function since it is usually influenced by other factors such as blood flow, pre- or post-load, or by animal training conditions. Additionally, it might result in death of the animal due to heart failure, making follow-up studies impossible. Also, fluorescence-based measurements, while simple and inexpensive, depend on the use of mutant and transgenic lines, and thus the direct analysis of different strains is challenging. Further, the degree of sensitivity needs to be assessed by comparing uninjured and injured animals.

The most established and widespread method used to assess cardiac function and to monitor alterations upon MI is based on echocardiography [17]. There are numerous variables that can be measured by echocardiography as an expression of systolic function of the heart. The most useful expression of global ventricular function is the ejection fraction (EF). The EF is a simple measure of how much of the end-diastolic volume, the amount of blood present in the ventricle just before the following contraction, is pumped out of the ventricle with each contraction. Quantitatively, EF can be calculated from M-mode, 2D or 3D echocardiograms. Because 2D echocardiography permits visualization of the entire ventricle perimeter, it is superior to the one-dimensional M-mode approach for the measurement of cardiac chamber volumes and ejection fraction. Numerous methods have been applied to calculate ventricle volumes by 2D echocardiography. Currently, the most popular method is the biplane Simpson method. This method derives measurements by a formula that assumes ventricle parallel planes that are divided into a number of small segments. In individuals with uniform contractility or when only one apical view can be obtained, the left ventricular volume measured with a single plane or with area-length method is very similar [18]. The ventricular endocardial edge is traced from one apical view to create multiple cylinders whose volumes are summated to provide the ventricle volume.

In the zebrafish, echocardiography has been used to visualize cardiac function in the adult [19], [20] and to study the transitory functional impairment triggered by hypoxia-reperfusion of the whole animal [21]. We describe here a quantitative method based on 2D echocardiography to measure ventricular dysfunction upon severe and local cardiac injury, and to assess functional recovery during regeneration.

In contrast to other injury protocols, cryoinjury induces local necrotic and apoptotic cell death, mimicking the conditions triggered by ischemic damage in humans. Also, analogous to the human scenario, damaged tissue is gradually substituted by fibrotic tissue. Despite this accumulation of fibrotic tissue, the zebrafish is able to gradually replace scar tissue with newly formed cardiac tissue [8]. While regeneration was completed in the long term, signs of ventricular remodeling, such as ventricular enlargement and impaired myocardial contraction, could be observed. We thus explored in more detail the recovery of ventricular function upon cryoinjury, using newly-developed quantitative echocardiographic methods to monitor and calculate the global ventricular contractility fraction and segmental wall motion. Our results demonstrate that while adult zebrafish exhibit impaired contractility in some segments of the ventricular wall, the global ventricular function recovers its normal pumping performance after cryoinjury.

## Methods

### Fish

All experiments were conducted with adult zebrafish aged 6–18 months and ranging from 2 to 4 cm in length and 250 to 500 mg in weight raised at 3 fish·l^−1^. For studies involving temporal evaluation of the cardiac function, fish were individually maintained in small 0.8 l tanks. The same number of male and female fish was used in all experiments. Fish lines used were the wild-type AB strain (ZIRC, Eugene, OR, USA), Tg(*mpeg1*:*Gal4*)/(*UAS*:*GFP*)[22], [23] and Tg(*myl7:nucDsRed*) [24].

### Ethics

Animal procedures conformed to Spanish bioethical regulations (Real Decreto 53/2013). The ethics committee that approved this study was the Community of Madrid, “Dirección General de Medio Ambiente”, “Conserjería de Medio ambiente, Vivienda y Ordenación de Territorio”.

### Surgery

Ventricular cryoinjury or sham operations were performed as previously described [25].

### Echocardiography

Animals were anaesthetized by immersion for approx. 5 min in a combined solution of 60 µM tricaine/3 mM isoflurane dissolved in fish tank water [26]. Immediately after detecting complete anaesthesia, by gently squeezing the caudal fin, the fish were placed ventral side upwards into a foam holder for immobilization. Measurements were performed using the VEVO 2100 system (VisualSonics) coupled to a 50 MHz ultrasound probe. The transducer was attached to a mechanical holder and lowered into the solution with a 3 mm clearing distance to the fish skin. The animal was rotated at different positions to obtain a plane where the ventricle apex was optimally visualized, obtaining a true ventricle long axis. The transducer was positioned over the pericardial sac, which could be easily identified by its position between the pectoral fins, its silvery pigmentation and the beating heart that can be appreciated underneath. The transducer was scanned over a sector-shaped area of 4 mm by 4 mm at a frame rate up to 200 images per second. The high frame capability facilitated the visualization of heart wall and valve motion, and improved the accuracy of measurements of heart chamber borders and their variations over the heart cycle. The whole imaging procedure and image analysis took 10 minutes.

### Histological processing, *in situ* hybridization and immunofluorescence

Hearts were fixed in 4% paraformaldehyde (PFA) in phosphate-buffered saline (PBS) overnight at 4°C. When used for *in situ* hybridization, samples were processed as described [8]. *In situ* hybridization on paraffin sections was performed according to Mallo et al. with some modifications [27]. A probe for the natriuretic peptide encoding gene, *nppa,* was cloned from cDNA using the following primers: For: 5′-ACACGTTGAGCAGACACAGC-3′; Rev: 5′-TGTTAACAAATTAAGCCGTATTGT-3′ as reported [28].

To detect nucDsRed protein, samples were equilibrated in 30% sucrose, cryosectioned and immunostained using a rabbit anti-DsRed antibody (Living Colors, Clontech). An Alexa-568-conjugated secondary antibody (Invitrogen) was used to reveal primary antibody signal. Nuclei were stained with DAPI and slides were mounted using Vectashield (Vector). Acid fuchsin-orange G (AFOG) stain was used to detect connective tissue (fibrin, red; collagen, blue).

### Imaging and video processing

Nikon Eclipse 90i and Olympus BX51 microscopes were used to image histological sections and a Leica TCS SP-5 confocal microscope was used for immunofluorescence imaging. Videos were exported as uncompressed files and processed in Fiji: videos were cropped to reduce file size and labels added. Processed videos were saved at the same frame rate than acquisition, 40 frames per second (fps), in JPEG compressed format.

### Quantitative real-time (qRT) PCR

RNA from cardiac ventricles was extracted using 0.5 mL Trizol reagent (Ambion, Life Technologies). 1 ventricle was used per biological replicate. Between 4 and 5 biological replicates were analyzed per time point. RNA was transcribed to cDNA using the High-Capacity cDNA Reverse Transcription Kit (Applied Biosystems, Life Technologies). qRT-PCR was performed using Power SYBR Green PCR Master Mix (Applied Biosystems, Life Technologies) and normalizing *nppa* expression with the geometric mean of the expression level of two constitutive genes: *EF1-alpha* and *rps11*. The following primers were used: *nppa*Forward 5′ CAAGCGCACGCGTTGA 3′, *nppa*Reverse 5′ TCTTGAGCTTGGCCATGTTG 3′ *EF1-alpha*Forward 5′ CAGCTGATCGTTGGAGTCAA 3′, *EF1-alpha*Reverse 5′ TGTATGCGCTGACTTCCTTG 3′, *rps11*Forward: 5′ GATGGCGGACACTCAGAAC 3′, *rps11*Reverse: 5′ CCAATCCAACGTTTCTGTGA 3′.

### Statistical Analysis

Differences between mean values of experimental groups were tested for statistical significance by one-way ANOVA followed by Tukey's honest significant difference test to control for the multiplicity of the tests. Model assumptions of normality and homogeneity of variance were checked with conventional residual plots. We did not observe any strong deviation from normality or heterogeneity of variance that would justify the use of non-parametric tests. Data on the wall motion score index (WMSI) at 140 dpi were analyzed using the Wilcoxon Signed Rank Test comparing to a theoretical median of 1. Data on myocardial density were analyzed for statistical significance by two-tailed Student's t-test. Statistical significance was assigned at P<0.05.

## Results

### Cryoinjury leads to a transient functional impairment of the cardiac ventricle

**Figure 1:**
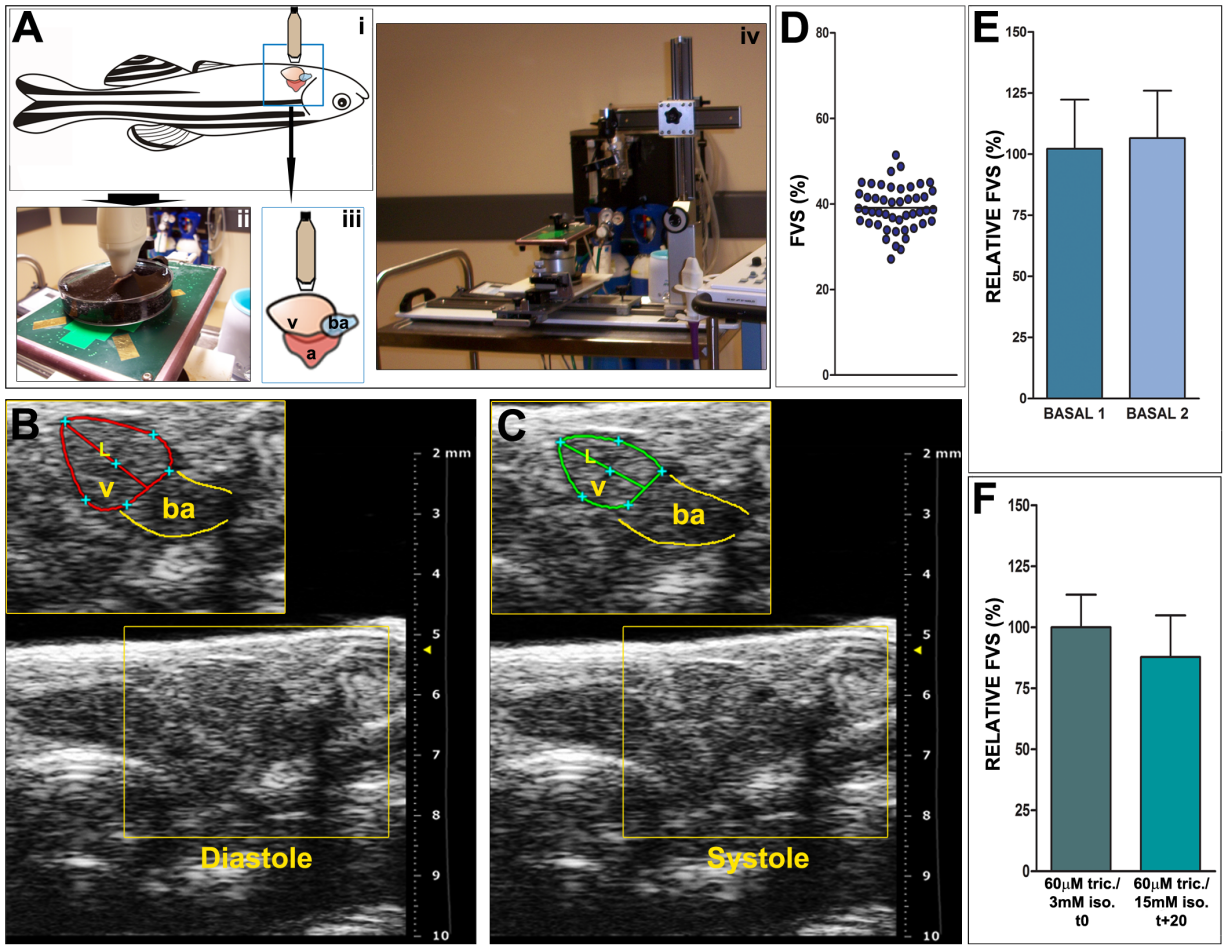
Echocardiographic image acquisition and basal fractional volume shortening (FVS) quantification. (A) Schematic representation of animal positioning for image acquisition and picture of the set up. (i) Animals are positioned ventrally and (ii) are immobilized in the same way as for surgical procedures, in a Petri dish, and are covered with fish water containing anaesthetic solution. This positioning allows for a transducer to be placed directly over the body wall at the level of the heart (iii). The transducer is attached to a holder to allow a stable position during acquisition (iv). **(B,C)** Details from representative 2D echocardiography images from an uninjured zebrafish heart showing maximal ventricular dilatation (B, diastole) and maximal ventricular contraction (C, systole). The diastolic (red) and systolic (green) ventricular areas are outlined and the length of the apical image long axis is also indicated (L). Red and green lines in B and C highlight ventricular border in diastole and systole, respectively. Yellow lines indicate the bulbus arteriosus (BA). **(D)** FVS obtained in basal conditions (n = 47, mean ± SD  =  39 ± 5). **(E)** Comparison of FVS in basal conditions at two different days, with an interval of 7 days. Shown are means ± SD. The relative FVS (RFVS) of BASAL2 versus BASAL1 within the same animal are statistically comparable (p = 0.1099, Wilcoxon matched-pairs signed rank test). **(F)** FVS measured in basal conditions with different dosages of anesthesia and throughout time in the same animal. Initial anesthesia conditions are the same as for all acquisitions (60 µM tricaine/3 mM isoflurane). The final acquisition was taken 20 minutes later and the final anesthesia dose was 60 µM tricaine/15 mM isoflurane. Differences in the average FVS are not statistically significant (*p* = 0.1094, Wilcoxon matched-pairs signed rank test). A, atrium; ba, bulbus arteriosus; FVS, fractional volume shortening; L, length of the apical image long axis; RFVS, relative fractional volume shortening; v, ventricle.

In contrast to the human and mouse, which present a well-defined ventricular cavity, the zebrafish ventricle wall is formed by a highly trabeculated subendocardium and a thin compacted subepicardial layer [29]. This highly-trabeculated myocardium prohibits an accurate determination of the endocardial border. Thus, we devised a protocol using an area-length method, but in contrast to studies in humans, we outlined the epicardial edge rather than the endocardial edge (Fig. 1A–C). The diastolic and systolic lengths of the apical image long axis (L) and its diastolic and systolic area (A) were measured (Fig. 1B,C). Diastolic and systolic left ventricular volumes (V) were assessed prospectively using the algorithm:

V  =  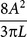


The fractional volume shortening (FVS) was calculated according to:

FVS =  100 × 
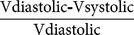


Using this method, we have determined that the average FVS in adult zebrafish is 39±5% in uninjured animals (n = 47, Fig. 1D, S1 Movie and S2 Movie).

Before applying the cryoinjury, we first sought to determine the variability in echocardiography measurements taken at different days, in order to establish basal physiological changes in cardiac function. FVS of 17 fish was measured on two different days, with an interval of 7 days. We found that in untreated zebrafish, the relative fractional volume shortening (RFVS) varied ± 20% (Fig. 1E). In order to test the robustness of the measurements, we challenged the system, keeping the fish for a longer period and with higher concentrations of anesthesia (n = 8). The control group was measured as described in Materials and Methods. This group was compared with a second group of fish treated with an excess of anesthesia, in which fresh isoflurane was added every five minutes to the anesthetic solution in which the fish were submerged. When the final measurement was taken, fish had been under anaesthesia for a total of 25 minutes and concentration of anesthetics was 60 µM tricaine/15 mM isoflurane. No significant differences in FVS were found between initial (t0) and final (t+20) measurements (Fig. 1F). From these control experiments, we concluded that in adult zebrafish, variations on the RFVS of 20% were within the normal physiological range.

**Figure 2:**
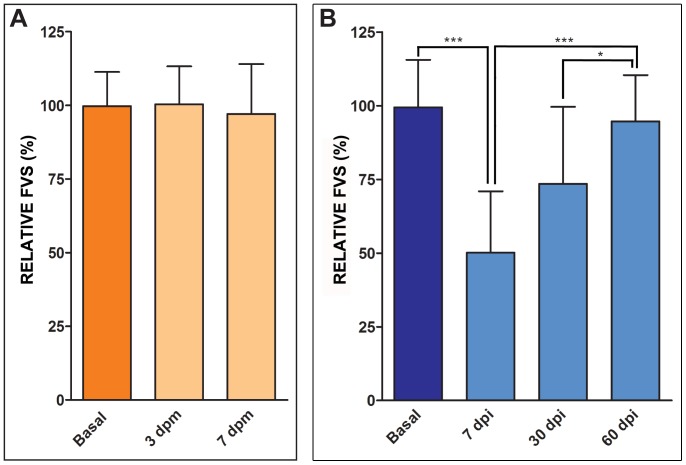
Cryoinjury transiently impairs ventricular pumping efficiency. (A,B) Temporal evolution of changes in the relative FVS in sham (A) and cryoinjured (B) animals in a longitudinal study. Graphs represent relative mean values and SD. **(A)** The relative FVS (RFVS) is not significantly changed in animals after sham operation at 3 (n = 14) or 7 (n = 21) dpm (*p* = 0.884; one-way ANOVA). **(B)** Cryoinjured animals show a temporal decrease in the RFVS of 50%, which is gradually recovered around 60 dpi (*** p<0.001; ** p<0.01; * p<0.05; one-way ANOVA followed by Tukey's honest significant difference test, n = 31). dpi, days postinjury; dpm, days postmanipulation; FVS, fractional volume shortening; RFVS, relative fractional volume shortening.

We also tested if sham operations, consisting of pericardial wall opening, led to alterations in cardiac function. To do this, we recorded basal FVS of 6–18-month-old zebrafish (n = 21), and repeated the measurement on these animals at 3 (n = 14) and 7 (n = 21) days post manipulation (dpm). In order to follow up the relative changes of ventricular function, each animal was individually followed throughout the protocol. RFVS was not significantly changed under these conditions (Fig. 2A, S3–S5 Movies).

Next, we tested whether cryoinjury impairs cardiac function in the zebrafish. As before, FVS was calculated from measurements performed prior to injury (S6 Movie) and at different periods post-injury on the same animals (n = 31). At 7 days post-injury (dpi), RFVS decreased dramatically to 50% of basal levels (Fig. 2B, S7 Movie). At 30 dpi, when a considerable regeneration of the myocardium could be observed by histology, the mean RFVS value increased relative to the 7 dpi value; however, this did not reach statistical significance (S8 Movie). In contrast, at 60 dpi, a time point when relatively few fibrotic fibers could be observed, the RFVS had significantly recovered and was comparable with the level obtained in basal conditions (S9 Movie). Thus, while sham operation did not affect cardiac function, cryoinjury led to a transient functional impairment, which recovered at 60 dpi.

### Echocardiography as a method to predict ventricular cryoinjury

**Figure 3:**
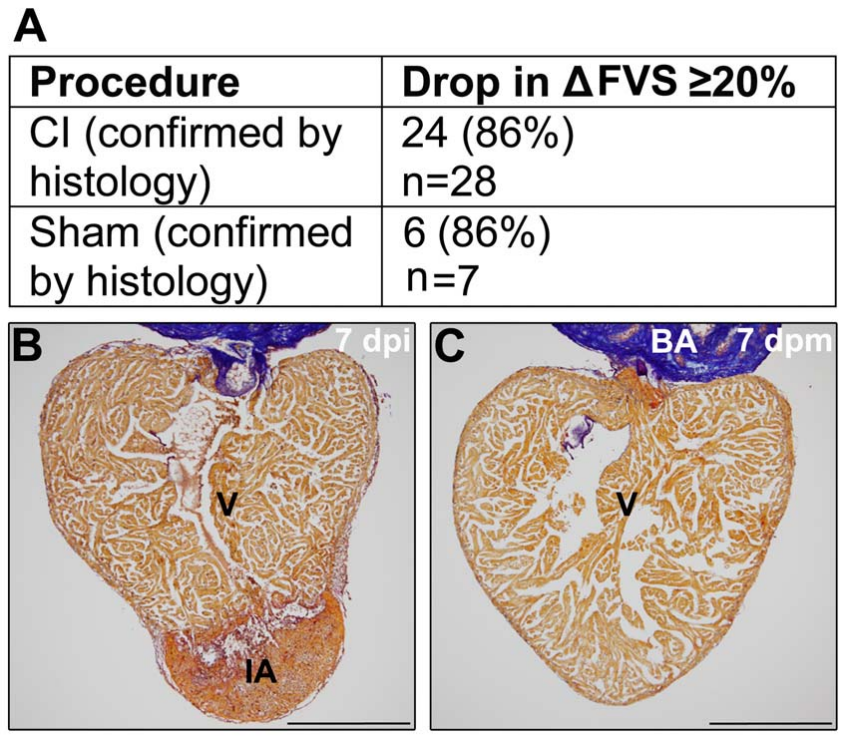
Correlation between histology of imaged hearts and echocardiographic analysis. **(A,B)** Groups of animals in which cryoinjury was confirmed by histological AFOG staining after echocardiography. In 24 out of 28 fish, cryoinjury was diagnosed at 7 dpi by measurement of a drop of the RFVS ≥ 20% compared to the equivalent basal measurement. Only one from 7 sham-operated fish presented a drop in RFVS ≥ 20%. (**C**) Subsequent histological staining however did not support an alteration in the cardiac morphology or injury in any of the sham operated animals. BA, bulbus arteriosus; IA, injured area; dpi, days postinjury; dpm, days postmanipulation; V, ventricle. Size bars, 200 µm.

In order to test the applicability of echocardiography to predict ventricular injury, measurements were performed blindly on sham operated and cryoinjured animals. During echocardiography, we additionally qualitatively evaluated pumping efficiency. Hearts of fish revealing impaired cardiac function by 2D echocardiography were scored as cryoinjured. After echocardiography, the fish were sacrificed and fixed for histology. RFVS was then analyzed and compared to histological preparations. We found that, in the majority of cases, there was a good correlation between echocardiographic measurements and detection of injury by histology. From 28 animals that were cryoinjured and in which a lesion was detected by histology, we could detect a decline in RFVS >20% in 24 animals by echocardiography (Fig. 3A–B). In the remainder, the RFVS did not change, however an injury was visible upon histological analysis (Fig. 3C). In sham-operated fish, we observed that in only 1 out of 7 cases the RFVS decreased to a value comparable with that observed in injured animals (Fig. 3A, C). The false positive could be successfully eliminated if, in addition to the RFVS measurements, the qualitative evaluation performed in 2D was also considered (Fig. 3A). One explanation for these false positive/negative results could be that a change of overall cardiac morphology or heart rotation occurred between measurements during the longitudinal study in a minority of analyzed fish. As a consequence, the ventricular diameter would have been calculated at a different angle in the two measurements and the assumption of a constant ventricular volume would lead to an unrealistic measurement. It is also possible that in the case of false negatives, while one part of the wall is affected by the injury and not functioning correctly, the remaining ventricular wall can compensate for this segment. Nonetheless, application of the Coheńs kappa coefficient statistical test demonstrated a good correlation between echocardiographic measurements and detection of injury by histology (Cohen's kappa coefficient: 0.62, p<0.0002).

### Cryoinjury leads to long-term alteration of ventricular wall motion

We had previously observed that while fibrotic tissue regression was nearly complete at 60 dpi, freshly-dissected hearts revealed morphological alterations such as a balloon-shaped ventricle, thickened myocardial wall and irregular contraction [8]. In order to quantify the observations in vivo, we used echocardiography to assess regional ventricular wall motion.

Wall motion score index, based on protocols by Schiller and colleagues [30], is a semi-quantitative analysis of regional systolic function used to assess human cardiac function. Accordingly, the left ventricle is divided into several segments, and a numeric score is assigned to each segment depending on its contractility characteristics. If the segment moves correctly, it is scored as 1. Higher scores indicate more severe wall-motion abnormalities, ranging from akinesia (no wall motion) to dyskinesia (irregular and uncoordinated motion) since an irregular and aberrant wall motion has more negative consequences for overall cardiac function than the lack of function of an individual segment. The wall motion score index (WMSI) represents the extent of regional wall motion abnormalities.

**Figure 4:**
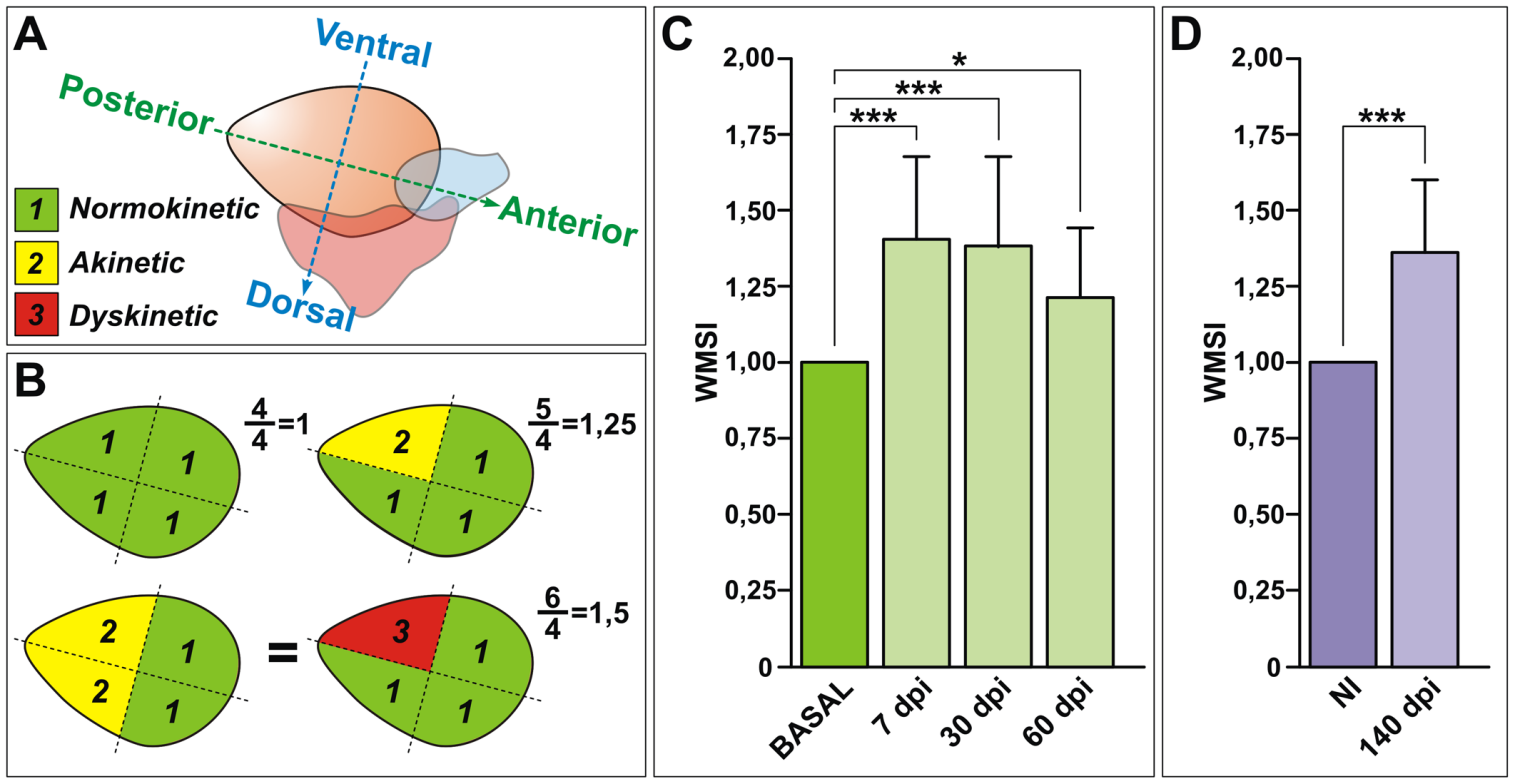
Ventricular wall motion is not fully recovered after cryoinjury. **(A)** Schematic representation of segmental criteria of the zebrafish ventricle, considering the antero-posterior and dorso-ventral axis. Depending on their motility, segments are scored as normokinetic (“1”), akinetic (“2”) or dyskinetic (“3”). **(B)** Theoretical representations of the wall motion score index (WMSI) showing ventricles from healthy controls (WMSI = 1) and cryoinjured animals (WMSI>1). **(C)** Temporal evolution of changes in the WMSI in cryoinjured animals in a longitudinal study. After injury, the WMSI increased and remained elevated even at 60 dpi, indicating that wall motion is affected. Graphs represent mean values and SD (*** p<0.001; * p<0.05; one-way ANOVA followed by Tukey's honest significant difference test, n = 20). (**D**) The WMSI is not recovered at extended stages of regeneration (n = 17), and it is not affected in siblings (NI) of the same age (n = 3). *** *p* = 0.00009, Wilcoxon Signed Rank Test comparing to a theoretical mean of 1. dpi, days postinjury; NI, not injured; WMSI, wall motion score index.

Because of its reduced size, we divided the zebrafish ventricle into four segments only according to the ventrodorsal and anteroposterior position (Fig. 4A). Each segment was analyzed individually and scored on the basis of its epicardial motion. This score is a 3-level score (from normal to the most pathological) defined as follows:

- score 1  =  normokinesia (normal epicardial motion of the myocardial segment, S10 Movie)
- score 2  =  akinesia (absence of epicardial motion, S11 Movie)
- score 3  =  dyskinesia (paradoxical systolic epicardial motion, S12 Movie)

Wall motion score index is derived as a sum of all scores divided by the number of segments visualized. Thus an index equal to 1 implies a normal contractility in all segments and an index greater than 1 implies that there are segments with abnormal contractility (Fig. 4B). We analyzed the wall motion in sham (n = 20) and cryoinjured (n = 20) animals in a longitudinal study. As expected, prior to injury the WMSI was 1 (Fig. 4C). This was also the case for sham animals at all stages analyzed (not shown). At 7 dpi, the WMSI was close to 1.5, indicating impaired segmental wall motion. Wall motion did not recover completely at 30 or 60 dpi (Fig. 4C). These results suggest that at the regenerative stage, when the global ventricular pumping performance is recovered, the injured walls in some cases still show significant motion defects.

To test if the ventricular segmental wall motion recovered at advanced stages of regeneration, we analyzed animals at 140 dpi, a period when histology revealed complete scar removal and structural restoration [8]. While uninjured siblings of equivalent age showed normal contractility of all segments (n = 3), ventricular wall motion was not completely recovered in cryoinjured animals even at these extended post-injury stages (n = 17, Fig. 4D).

Taken together, these findings indicate that while pumping efficiency is recovered rapidly after cryoinjury, ventricular wall motion remains altered even at prolonged stages post-injury. Since there is complete histological regeneration, these findings suggest that cardiac muscle structure or maturation is not entirely restored upon cryoinjury and might lag behind the functional recovery.

### The cryoinjured wall develops myocardial hyperplasia

**Figure 5:**
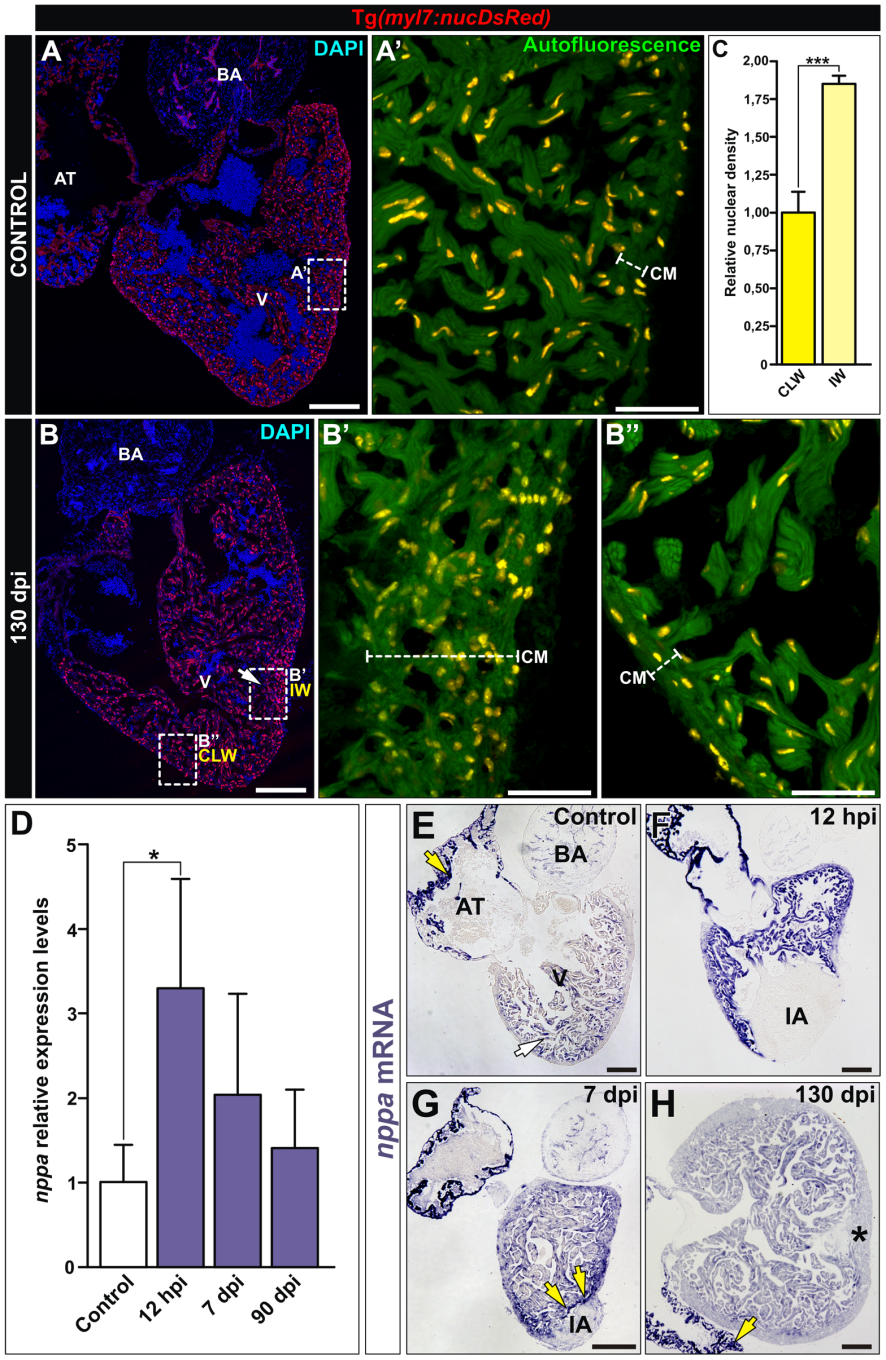
Cryoinjury induces local, long-term alterations in myocardial organization. **(A,B)** Immunohistochemistry on sagittal sections of control (A,A’) and cryoinjured (B-B”) hearts at 130 dpi from the Tg(*myl7:nucDsRed*) line. A’–B” are zoomed images of boxed areas in A and B, additionally showing autofluorescence to reveal tissue organization. **(A-A’)** In control hearts, one or two cells constitute the thickness of the compact myocardium (CM). **(B-B”)** At 130 dpi, the injured wall (IW) shows an abnormal increase in the number and distribution of cardiomyocytes compared with the contralateral wall (CLW). **(C)** Quantification of the nuclear density relative to the compact tissue reveals an increase in cardiomyocyte density in the IW compared to the CLW. Graph represents mean values and SD (*** p = 0.006, two tailed Student's t-test; 100–150 cells counted per section, 3 sections per heart, n = 3 animals analyzed). **(D)** qPCR from ventricular RNA samples reveal induction of the natriuretic peptide encoding gene *nppa* upon cryoinjury. Graph represents mean values and SD, n  =  4-5 replicates, Expressions levels were normalized to that of *ef1α* and *rps11* and further normalized to that of the uninjured sample. (* p<0.05; one-way ANOVA followed by Tukey's honest significant difference test). **(E-H)** Sections of cryoinjured hearts at the indicated times post-injury hybridized with a riboprobe for *nppa* mRNA. Yellow arrows mark areas of strong *nppa* expression. **(E)** In control hearts, *nppa* is highly expressed in the atrium (yellow arrow) and at lower levels in the trabecular myocardium (white arrow). **(F-G)** Shortly after injury, *nppa* is strongly upregulated in the ventricular myocardium. **(H)** At 90 dpi, the levels of *nppa* expression are similar to those detected in control hearts. Observe the increase in thickness of the compact layer of the injured wall (asterisk) revealed by no expression of *nppa*. AT, atrium; BA, bulbus arteriosus, CLW, contralateral wall; CM, compact myocardium; hpi, hours postinjury; dpi, days postinjury; IA, injured area; IW, injured wall; V, ventricle. Bars, 200 µm (full views), 50µm (magnifications).

To gain insight into the causes of this local motility defect, we assessed cardiomyocyte density in the region of injury. In comparison with control animals and contralateral, uninjured walls, cardiomyocyte nuclei were densely packed in injured ventricular walls (Fig. 5A–B”). Quantification of nuclei revealed an almost 2-fold increase in cardiomyocyte nuclei per area in the regenerated ventricular wall (Fig. 5C).

To test if the abnormal organization of the injured wall was sensed as increased wall tension, we analyzed the expression of *nppa*. While *nppa* is expressed during embryonic development in the forming trabecular myocardium [31], it is also reactivated in the adult heart upon stress [32]. *nppa* expression was analyzed by qRT-PCR in cryoinjured ventricles. Cryoinjury led to an increase in *nppa* expression in ventricles at 12 hours postinjury (hpi) (Fig 5D). Expression of *nppa* peaked at 12 hpi, and at 7 dpi the differences to controls were no longer statistically significant, suggesting a downregulation of *nppa* from this time point of regeneration onwards. In addition, mRNA in situ hybridization was performed to characterize the expression pattern of *nppa* in the regenerating heart. We observed that uninjured adult hearts exhibited low levels of *nppa* in the ventricle (Fig. 5E, n = 3), with detectable staining restricted to the trabecular myocardium, as previously reported during mammalian development [33]. As expected, cryoinjury induced expression of *nppa* at 12 hpi (n = 2, Fig. 5F). In good agreement with the results from PCR, *nppa* expression remained high at 7 dpi compared with control (n = 2; Fig. 5G), and decreased to basal levels at 90 dpi (n = 2, Fig. 5H). Of note, the thickened myocardial wall was devoid of *nppa* expression. A possible interpretation is that this *nppa*-negative area represents an expansion of the cortical myocardial layer during cardiac regeneration. Upregulation of *nppa* expression could be the result of a transient activation of the developmental gene program, as has been shown to occur in the initial stages of cardiomyocyte regeneration [34] or perhaps it might reflect a stress response until reestablishment of proper cardiac function.

## Discussion

We have established a non-invasive protocol to assess ventricular function in zebrafish in vivo. The echocardiography parameters chosen are based on those used in clinical practice and are modified for the small and hypertrabeculated ventricle of the zebrafish.

It should be noted that inter- and intraspecimen variability of the RFVS in zebrafish was approximately 20%. This is two times higher than the variability reported for humans and mouse models [35], [36] and might likely be a consequence of more inaccurate measurements due to the small size of the zebrafish heart. Similar to the standard procedures in the clinic, we strongly recommend that all the measurements in a study should be performed by the same observer. This reduces interobserver variability and improves reproducibility. Clearly, non-experts will require some training before they can become proficient at performing reproducible echocardiographic measurements on zebrafish, especially with regards to visualizing the epicardial borders of the heart and positioning the probe for recording.

Given that the zebrafish represents a small vertebrate animal that is gaining importance as a model of cardiac regeneration, we envisage that these echocardiographic protocols for quantification of global and segmental ventricular function will be very valuable to help assess cardiac regeneration in vivo, and in the study of genetic gain and loss of function models and also the effect of chemical compounds for drug discovery. The main advantages of this method are the direct application to any zebrafish strain and the ability for longitudinal monitoring of the same animals in a non-invasive manner.

We observed that although complete recovery of the ventricular pumping efficiency was observed at 60 dpi, contraction of some ventricular wall segments was not fully restored, even after extended periods. These results are consistent with our previous observations of altered morphology in the regenerated heart, i.e. the increase in thickness of the compact layer and the morphological changes in ventricular geometry [8]. Together they are indicative of compensatory mechanisms to provide proper function despite the inefficient contraction of ventricular wall segments. It should be noted that, during surgery, pericardial adhesions often appear between the body wall and the dorsal wall of the ventricle. While these adhesions might affect proper wall motion, our previous observations of an improper heart contraction in dissected hearts at 130 dpi do not fully support this possibility. However, overt ventricular remodeling is unlikely to occur, as we have not observed scarring in remote areas even at 130 dpi [8], or up-regulation of marker genes such as *nppa*. The notable increase in myocardial cell nuclei observed at the injured ventricular wall is indicative of local hyperplasia, which could also interfere with normal contraction. It will be important to assess if the higher density of cardiomyocyte nuclei found after cryoinjury correspond to immature or binucleated cardiomoycytes. Nonetheless, the global cardiac function is normal, suggesting a capacity for the zebrafish heart to compensate for the presence of akinetic regions by increasing the pumping capacity of the remainder of the ventricular wall. In sum, our observations underscore the need for a more refined phenotypic analysis of heart injuries, including functional studies, and demonstrate that echocardiographic measurements can be useful tools to allow a correct interpretation of results on cardiac regeneration in the zebrafish.

## Supporting Information

S1 Movie **2D-Echocardiography of an uninjured zebrafish heart.** Shown is a sagittal section, the head of the fish is to the right, ventral side is upwards (see Fig. 1A for orientation). The epicardial border of the ventricle is marked in magenta. The movie is acquired at 48 Hz.
(MOV)

S2 Movie **2D-Echocardiography at 7 days post cryoinjury.** Shown is the same fish as in Movie S1. The asterisk marks the injured ventricular apex. Shown is a sagittal section, the head of the fish is to the right, ventral side is upwards (see Fig. 1A for orientation). The epicardial border of the ventricle is marked in magenta. The movie is acquired at 48 Hz.
(MOV)

S3 Movie **2D-Echocardiography before sham operation.** Shown is a sagittal section, the head of the fish is to the right, ventral side is upwards. Bulboventricular and atrioventricular valves are marked with arrowheads. The movie is acquired at 48 Hz. at, atrium; ba, bulbus arteriosus; V, ventricle.
(AVI)

S4 Movie **2D-Echocardiography at 3 days postmanipulation.** Shown is a sagittal section of the same fish shown in Movie S3, 3 days upon opening the pericardial cavity (sham operation, arrow). The head of the fish is to the right, ventral side is upwards. The movie is acquired at 48 Hz. dpm, days postmanipulation; V, ventricle.
(AVI)

S5 Movie **2D-Echocardiography at 7 days postmanipulation.** Shown is a sagittal section of the same fish shown in Movies S3 and S4, 7 days upon opening the pericardial cavity (sham operation). The head of the fish is to the right, ventral side is upwards. The movie is acquired at 48 Hz. dpm, days postmanipulation; V, ventricle.
(AVI)

S6 Movie **2D-Echocardiography before cryoinjury.** Shown is a sagittal section, the head of the fish is to the right, ventral side is upwards. The movie is acquired at 48 Hz. V, ventricle.
(AVI)

S7 Movie **2D-Echocardiography 7 days postinjury.** Shown is a sagittal section of the same fish shown in Movie S6, at 7 days after cryoinjury of the ventricular apex (asterisk). The head of the fish is to the right, ventral side is upwards. The movie is acquired at 48 Hz. dpi, days postinjury; V, ventricle.
(AVI)

S8 Movie **2D-Echocardiography 30 days postinjury.** Shown is a sagittal section of the same fish shown in Movies S6 and S7, at 30 days after cryoinjury of the ventricular apex (asterisk). The head of the fish is to the right, ventral side is upwards. The movie is acquired at 48 Hz. dpi, days postinjury; V, ventricle.
(AVI)

S9 Movie **2D-Echocardiography 60 days postinjury.** Shown is a sagittal section of the same fish shown in Movies S6–8, at 60 days after cryoinjury of the ventricular apex (asterisk). The head of the fish is to the right, ventral side is upwards. The movie is acquired at 48 Hz. dpi, days postinjury; V, ventricle.
(AVI)

S10 Movie **Example of normokinesia.** Shown is a sagittal section of a fish with its head to the right, ventral side is upwards. The epicardial border is outlined and the ventricle divided into 4 segments. A score of 1 indicated normal motion of the epicardial borders of that segment. The movie is acquired at 48 Hz. Two acquisitions of a few seconds have been concatenated to allow a better visualization of the ventricular wall motion. V, ventricle.
(AVI)

S11 Movie **Example of akinesia.** Shown is a sagittal section of a cryoinjured fish with its head to the right, ventral side is upwards. The epicardial border is outlined and the ventricle divided into 4 segments. A score of 2 indicates that there is no motion of the epicardial borders found in that segment (akinesia). The movie is acquired at 48 Hz. Two acquisitions of few seconds have been concatenated to allow a better visualization of the ventricular wall motion. V, ventricle.
(AVI)

S12 Movie **Example of akinesia.** Shown is a sagittal section of a cryoinjured fish with its head to the right, ventral side is upwards. The epicardial border is outlined and the ventricle divided into 4 segments. A score of 3 indicates that there is paradoxical motion of two neighboring segments (dyskinesia). The movie is acquired at 48 Hz. Two acquisitions of few seconds have been concatenated to allow a better visualization of the ventricular wall motion. V, ventricle.
(AVI)

## References

[1] Laflamme MA, Murry CE (2011) Heart regeneration. Nature 473:326–335. 21593865 10.1038/nature10147 PMC4091722

[2] Sedmera D, Thompson RP (2011) Myocyte proliferation in the developing heart. Dev Dyn 240:1322–1334. 21538685 10.1002/dvdy.22650 PMC3271704

[3] Jennings RB, Murry CE, Steenbergen C Jr, Reimer KA (1990) Development of cell injury in sustained acute ischemia. Circulation 82:II2–12. 2394018

[4] Choi WY, Poss KD (2012) Cardiac regeneration. Curr Top Dev Biol 100:319–344. 22449849 10.1016/B978-0-12-387786-4.00010-5 PMC3342383

[5] Raya A, Koth CM, Buscher D, Kawakami Y, Itoh T, et al (2003) Activation of Notch signaling pathway precedes heart regeneration in zebrafish. Proc Natl Acad Sci U S A 100 Suppl 1 11889–11895. 12909711 10.1073/pnas.1834204100 PMC304103

[6] Poss KD, Wilson LG, Keating MT (2002) Heart regeneration in zebrafish. Science 298:2188–2190. 12481136 10.1126/science.1077857

[7] Chablais F, Veit J, Rainer G, Jazwinska A (2011) The zebrafish heart regenerates after cryoinjury-induced myocardial infarction. BMC Dev Biol 11:21. 21473762 10.1186/1471-213X-11-21 PMC3078894

[8] Gonzalez-Rosa JM, Martin V, Peralta M, Torres M, Mercader N (2011) Extensive scar formation and regression during heart regeneration after cryoinjury in zebrafish. Development 138:1663–1674. 21429987 10.1242/dev.060897

[9] Schnabel K, Wu CC, Kurth T, Weidinger G (2011) Regeneration of cryoinjury induced necrotic heart lesions in zebrafish is associated with epicardial activation and cardiomyocyte proliferation. PLoS One 6:e18503. 21533269 10.1371/journal.pone.0018503 PMC3075262

[10] Wang J, Panakova D, Kikuchi K, Holdway JE, Gemberling M, et al (2011) The regenerative capacity of zebrafish reverses cardiac failure caused by genetic cardiomyocyte depletion. Development 138:3421–3430. 21752928 10.1242/dev.068601 PMC3143562

[11] Kikuchi K, Holdway JE, Werdich AA, Anderson RM, Fang Y, et al (2010) Primary contribution to zebrafish heart regeneration by gata4(+) cardiomyocytes. Nature 464:601–605. 20336144 10.1038/nature08804 PMC3040215

[12] Yu F, Li R, Parks E, Takabe W, Hsiai TK (2010) Electrocardiogram signals to assess zebrafish heart regeneration: implication of long QT intervals. Ann Biomed Eng 38:2346–2357. 20221900 10.1007/s10439-010-9993-6 PMC3117900

[13] Cao H, Yu F, Zhao Y, Zhang X, Tai J, et al. (2014) Wearable multi-channel microelectrode membranes for elucidating electrophysiological phenotypes of injured myocardium. Integr Biol (Camb). 10.1039/c4ib00052h PMC4124744 24945366

[14] Hoage T, Ding Y, Xu X (2012) Quantifying cardiac functions in embryonic and adult zebrafish. Methods Mol Biol 843:11–20. 22222517 10.1007/978-1-61779-523-7_2 PMC3762588

[15] Lindekleiv H, Wilsgaard T, Macfarlane PW, Lochen ML (2012) QT interval and the risk of myocardial infarction and all-cause death: a cohort study. J Cardiovasc Electrophysiol 23:846–852. 22509793 10.1111/j.1540-8167.2012.02308.x

[16] De Sutter J, Tavernier R, Van De Wiele C, De Backer J, Kazmierczak J, et al (1999) QT dispersion is not related to infarct size or inducibility in patients with coronary artery disease and life threatening ventricular arrhythmias. Heart 81:533–538. 10212174 10.1136/hrt.81.5.533 PMC1729042

[17] Steeds RP (2011) Echocardiography: frontier imaging in cardiology. The British Journal of Radiology 84: 237–244. 10.1259/bjr/77730594 PMC3473911 22723531

[18] Lang RM, Bierig M, Devereux RB, Flachskampf FA, Foster E, et al (2006) Recommendations for chamber quantification. Eur J Echocardiogr 7:79–108. 16458610 10.1016/j.euje.2005.12.014

[19] Sun L, Lien CL, Xu X, Shung KK (2008) In vivo cardiac imaging of adult zebrafish using high frequency ultrasound (45–75 MHz). Ultrasound Med Biol 34:31–39. 17825980 10.1016/j.ultrasmedbio.2007.07.002 PMC2292109

[20] Liu TY, Lee PY, Huang CC, Sun L, Shung KK (2013) A study of the adult zebrafish ventricular function by retrospective Doppler-gated ultrahigh-frame-rate echocardiography. IEEE Trans Ultrason Ferroelectr Freq Control 60:1827–1837. 24658716 10.1109/TUFFC.2013.2769 PMC4091976

[21] Parente V, Balasso S, Pompilio G, Verduci L, Colombo GI, et al (2013) Hypoxia/reoxygenation cardiac injury and regeneration in zebrafish adult heart. PLoS One 8:e53748. 23341992 10.1371/journal.pone.0053748 PMC3547061

[22] Ellett F, Pase L, Hayman JW, Andrianopoulos A, Lieschke GJ (2011) mpeg1 promoter transgenes direct macrophage-lineage expression in zebrafish. Blood 117:e49–56. 21084707 10.1182/blood-2010-10-314120 PMC3056479

[23] Asakawa K, Suster ML, Mizusawa K, Nagayoshi S, Kotani T, et al (2008) Genetic dissection of neural circuits by Tol2 transposon-mediated Gal4 gene and enhancer trapping in zebrafish. Proc Natl Acad Sci U S A 105:1255–1260. 18202183 10.1073/pnas.0704963105 PMC2234125

[24] Mably JD, Mohideen MA, Burns CG, Chen JN, Fishman MC (2003) heart of glass regulates the concentric growth of the heart in zebrafish. Curr Biol 13:2138–2147. 14680629 10.1016/j.cub.2003.11.055

[25] Gonzalez-Rosa JM, Mercader N (2012) Cryoinjury as a myocardial infarction model for the study of cardiac regeneration in the zebrafish. Nat Protoc 7:782–788. 22461067 10.1038/nprot.2012.025

[26] Huang WC, Hsieh YS, Chen IH, Wang CH, Chang HW, et al (2010) Combined use of MS-222 (tricaine) and isoflurane extends anesthesia time and minimizes cardiac rhythm side effects in adult zebrafish. Zebrafish 7:297–304. 20807039 10.1089/zeb.2010.0653

[27] Mallo M, Schrewe H, Martin JF, Olson EN, Ohnemus S (2000) Assembling a functional tympanic membrane: signals from the external acoustic meatus coordinate development of the malleal manubrium. Development 127:4127–4136. 10976045 10.1242/dev.127.19.4127

[28] Berdougo E, Coleman H, Lee DH, Stainier DY, Yelon D (2003) Mutation of weak atrium/atrial myosin heavy chain disrupts atrial function and influences ventricular morphogenesis in zebrafish. Development 130:6121–6129. 14573521 10.1242/dev.00838

[29] Hu N, Yost HJ, Clark EB (2001) Cardiac morphology and blood pressure in the adult zebrafish. Anat Rec 264:1–12. 11505366 10.1002/ar.1111

[30] Schiller NB, Shah PM, Crawford M, DeMaria A, Devereux R, et al (1989) Recommendations for quantitation of the left ventricle by two-dimensional echocardiography. American Society of Echocardiography Committee on Standards, Subcommittee on Quantitation of Two-Dimensional Echocardiograms. J Am Soc Echocardiogr 2:358–367. 2698218 10.1016/s0894-7317(89)80014-8

[31] Houweling AC, van Borren MM, Moorman AF, Christoffels VM (2005) Expression and regulation of the atrial natriuretic factor encoding gene Nppa during development and disease. Cardiovasc Res 67:583–593. 16002056 10.1016/j.cardiores.2005.06.013

[32] Gupta V, Gemberling M, Karra R, Rosenfeld GE, Evans T, et al (2013) An injury-responsive gata4 program shapes the zebrafish cardiac ventricle. Curr Biol 23:1221–1227. 23791730 10.1016/j.cub.2013.05.028 PMC3759223

[33] Zeller R, Bloch KD, Williams BS, Arceci RJ, Seidman CE (1987) Localized expression of the atrial natriuretic factor gene during cardiac embryogenesis. Genes Dev 1:693–698. 2962900 10.1101/gad.1.7.693

[34] Lepilina A, Coon AN, Kikuchi K, Holdway JE, Roberts RW, et al (2006) A dynamic epicardial injury response supports progenitor cell activity during zebrafish heart regeneration. Cell 127:607–619. 17081981 10.1016/j.cell.2006.08.052

[35] Gordon EP, Schnittger I, Fitzgerald PJ, Williams P, Popp RL (1983) Reproducibility of left ventricular volumes by two-dimensional echocardiography. J Am Coll Cardiol 2:506–513. 6875114 10.1016/s0735-1097(83)80278-2

[36] Wasmeier GH, Melnychenko I, Voigt JU, Zimmermann WH, Eschenhagen T, et al (2007) Reproducibility of transthoracic echocardiography in small animals using clinical equipment. Coron Artery Dis 18:283–291. 17496492 10.1097/MCA.0b013e3280d5a7e3

